# Des Anévrysmes Et De Leur Traitement

**Published:** 1857-03

**Authors:** 


ART. III.—*Des Anévrysmes et de leur Traitement.* Par PAUL BROCA, Agrégé à la Faculté de Médecine de Paris, Chirurgien des Hôpitaux, etc. Ouvrage accompagné de figures intercalées dans le texte. Paris, 1856. 8vo. pp. 931.

"OF the profoundly scientific character and high practical value of the publications upon medicine, which are continually emanating from the continental press, none but they who have access to those productions of the master spirits of our art, in their original language, can form an adequate conception or correct estimate." So says Dr. Palmer, in the Preface to his "Pentaglot Dictionary," and, from our acquaintance with French authors, being fully of his opinion, we opened the work of M. Broca with the most favorable anticipations.

The extraordinary size of M. Broca's work must at once strike with astonishment. He explains it himself, in the preface, by the necessity he found himself under, of not only describing the new methods for the treatment of aneurism, but, moreover, of renewing from one end to the other the study of all the others. The three new methods are, indirect compression, galvano-puncture, and coagulating injections; which methods, so interesting, and so important, he says, have not found their place in classic treatises.

The principles, which have guided him in the composition of his work, are those that have guided Malgaigne in his; he has attempted, according to his own declaration, to do for aneurism what this eminent master has done for fractures and luxations. Malgaigne, in publishing his "Traité des Fractures et des Luxations," declared, that his object was to "fill a gap in French surgical literature." "England and Germany, in our day, count several treatises on fractures and luxations; and, perhaps, there is some room for astonishment that France should have remained behind."* M. Broca should have honestly said as much; he should have declared that he wished "to fill a gap in French surgical literature," and he would be sure to receive the credit due to one who has toiled in a good work. After the announcement of his intention to be guided by Malgaigne, we would, naturally, look for an atlas, such as the magnificent one accompanying the work of this author, and which throws upon numbers of questions the decisive light of pathological anatomy. In its place some fourteen woodcuts are printed with the text; those of any importance being taken from Hodgson. Guided by the principles of Malgaigne, he should also have terminated his work, as Malgaigne promised to terminate his, though he did not accomplish it, for some reason unknown to us, by a general bibliography of all the classical writers, who have treated of diseases of the arteries; as La Roche, of our own country, has done for yellow fever in his great work on that subject. M. Broca declares, in his preface, that he has paid special attention to questions of history and of bibliography; and he could therein have traced, as Malgaigne was to do, "the history of the art on this subject, followed the progress of ideas, the series of authors, the chronology of the great works, and of their principal editions." This bibliography would have been the more useful, for that of Erichsen, attached to his collection of authors who have written on aneurism, printed for the Sydenham Society, in 1844, does not extend farther than the end of the last century.

It may be well to quote this passage from the preface of M. Broca's work, as it will afford an idea of the spirit of the production. After stating that, at the commencement of his undertaking, he had intended only to describe the new methods, hoping to find the old sufficiently well treated of, he adds: "This hope was not realized; I was not slow in finding that

*Loc. cit. page v.

the clinical descriptions were swarming with gaps and errors; that many historical questions had been entirely neglected; and that the greater number of the rest had been studied insufficiently and inexactly; that the pathological physiology of aneurism did not exist, and that no one had seriously occupied himself with the mode of action of the different methods." In the preparation of the work he declares, that he has appealed to the experience of surgeons of every country. We do not profess to be able to do anything of the kind; but, from our acquaintance with the experience of surgeons in the language in which we are writing, we can fairly declare that, in the work of M. Broca, there is nothing of any value in regard to the pathological anatomy and treatment of aneurism, which may not be found therein. In regard to the historical questions, it would be impossible for any one who had not access to the large libraries of the French capital, to discuss them with him. We can only say, that a great deal of fault must be found with the spirit in which they are written. Lamartine says, somewhere, that, "When God has a great idea to give to humanity, he places it in the brain of a Frenchman." M. Broca tries to prove that this is true, also, of the smallest.

In order that the reader, accustomed to the works of Miller, Erichsen, &c., may understand, let us give some extracts from what we see in recent French books. Cruveilhier, in 1852, says: "Pathological anatomy having taught us that two methods of cure existed for aneurism, to wit, first, the definite cure by obliteration; secondly, the provisional cure by coagulation; it is very evident, that the first mode of cure should be preferred whenever it is possible, and, in this respect, modern surgery leaves nothing to desire, for it has pushed skill and boldness to the farthest limits in the application of ligatures,—the only means it possesses of obtaining the obliteration of the vessel."—(Traité d'Anat. Path. Gén., Vol. II, p. 801.)

Malgaigne, in his Operative Surgery, devotes but eighteen lines to compression, and says that it can be applied only to aneurisms of the popliteal artery, or of the crural artery, and that it acts by means of a mechanical compressor applied on the arterial trunk against the pubis. This is what he said in his edition of 1849, and in his last edition, 1853, he has not added one word. All that Nélaton says is very true, but it is far from sufficient. "Compression can be exercised by means of a tourniquet, but, above all, by the aid of the compressor of Dupuytren. The compressor should, if possible, only act upon the vessel and the point diametrically opposite, in order not to hinder the circulation in the veins of the limb. It hinders the course of the blood, but does not interrupt it entirely, as some persons have thought. The sac gradually contracts. It can be obliterated by the blood which coagulates in its interior. Besides, should this compression not succeed in obliterating the vessel, it impedes the circulation sufficiently to cause the collaterals to dilate,—a fortunate circumstance, should the ligature be necessary." (Elémens de Pathologie Chirurgicale, Paris, 1844, p. 459.) In the Compendium de Chirurgie Pratique, the second volume of which, or the one containing the article on aneurism, was published in Paris, in 1851, compression is spoken of in high terms, but the compressor of Dupuytren is the instrument recommended, and it is said to act in two ways: 1st, by obliterating the vessel in the spots compressed, and 2d, by favoring the coagulation of the blood in the sac, in consequence of the obstacle caused to the circulation. "The portion of that liquid which is stagnating in the aneurismal sac loses its fluidity, and is transformed into clots." (P. 104.)

French surgery, then, had not made one step in the treatment of aneurism since the time of Dupuytren, that is, in the principles upon which the treatment should be conducted. In the publication of his clinical lectures by the Sydenham Society, in 1854, at page 34, Dupuytren, speaking of a case he had cured by compression, says: "To what was the cure owing? Was it accomplished by obliteration of the femoral, as a consequence of adhesive inflammation produced by compression? Certainly not; for in both instances the pulsation of the artery could be traced to the opening in the adductor magnus muscle. It can only be referred to coagulation of the blood in the aneurismal sac, induced by rest, and the absence of impulse from behind." This is taken from a clinical lecture delivered in 1818, and Dupuytren regards the case as one of those lucky chances which occur occasionally in the course of a long practice, but which cannot be referred to as a precedent.

Those who are acquainted with the works usually recommended here to students, those of Erichsen and Miller, for instance, will see how necessary in France was the publication of a work on aneurisms. There was a great demand there for the information contained in M. Broca's book, or the information to be found in works published in the English language in the past twenty years.

The work is divided into two parts. The first, comprising 195 pages, treats of the pathology of aneurism; the second, of their treatment.

There is perhaps no disease in which, in every point, the smallest as well as the most important, there is such difference, such contrariety of opinion, as in aneurism. These differences commence with the very derivation of the word itself; its orthography is disputed; its signification varies, and the divisions of aneurism are as contradictory as possible. *Aneurysma* is derived by some from the particle *α* privative, and from *νευρον*, a nerve, which might be translated enervation; by others from the verb *εὐρύνω*, I dilate; and by others from the verb *εὐρηνειν*, said to signify, *to gush forth*, but which we have been unable to find in our lexicon. Some write aneurism, others aneurysm. To write it with an *i* is, according to M. Broca (p. 203), "an error due to illiterate barbers, who put for the first time surgery into a vulgar tongue."* According to Miller and the Compendium de Chirurgie Pratique, *true* aneurisms are those which are the result of disease, the cyst consisting of one or more of the arterial coats, yet undivided. The term *false*, on the contrary, denotes those aneurisms in which the arterial tunics have been wholly divided, either by wound or by ulceration from without, and form no part of the aneurismal cyst. With others, as Nélaton and Erichsen, an aneurism is *false* as soon as one of the coats is torn. A rupture of one or two coats, with dilatation of the rest, is called by many authors a *mixed* aneurism, by which they mean that it partakes both of the true and of the false; it is mixed internal or mixed external, in accordance with the coat that may be preserved. With some surgeons, an arterial wound, with diffused cellular hemorrhage, is a primitive false aneurism; and with circumscribed, a consecutive. M. Broca, by primitive, means that the aneurism was produced at the moment of the accident, and by consecutive, that it manifested itself at the expiration of some time, some weeks, or even years.

Nélaton, by the word aneurism, signifies "every tumor formed by arterial blood contained in a dilated artery, or escaped from the vessel, but always communicating with it, and penetrating or not into a vein." Cruveilhier declares that we ought not to consider as an aneurism a tumor formed by blood effused around an opened artery; aneurism, properly so called, belongs to the class of dilatations; diffused or circumscribed cellular hemorrhage belongs to solutions of continuity. "The truth of these reflections is proved by the denomination of *false* aneurism, under which traumatic aneurism is designated: suppress at least from pathological anatomy the expression of *false*, which seems to accuse it of inability to determine certain morbid lesions;" (loc. cit. vol. ii, p. 724.) Gerdy says of false aneurisms: "There is nothing false in all this but the language, and the language is false only because the ideas are confused." (Practical Surgery, vol. ii, p. 537.)

The definition of M. Broca does not suffice for all the necessities of practice, as does the definition of Nélaton, and at the same time it admits into the class of aneurisms other tumors of arterial origin, which should be excluded by a definition given on scientific principles. "Aneurysm is a circum-

* An orthography founded on etymology, though preferable on many accounts, is in many cases impossible, and the usual mode of writing should not be changed to suit a derivation. To write *y* for upsilon is very well, but custom writes oxide, and not oxyd, aneurism, and not aneurysm, and therefore they are correct. A strict adherence to etymology would cause much odd orthography; for instance, as in the Greek root the *r* is aspirated, we should spell rose *rhose*.

scribed tumor, full of blood, liquid or concrete, communicating directly with the canal of an artery, and limited by a membrane, bearing the name of sac." Though he has given a new definition, however, he does not abide by it, but virtually adopts that of Nélaton, for he does not reject from the class of aneurisms what are called *diffused* aneurisms. He does so because they only differ from others by one character, namely, the absence of a membranous wall. He therefore does not hold to the sac, the only point in which his definition differs from Nélaton's. He does very well not to abide by his definition; for bloody and pulsating tumors, not provided with a sac, at a certain period, can afterwards become encysted; it is often impossible, during life, to know if they have a membranous wall or not, and moreover, in both cases, the therapeutical indications are very often the same.

The classification of aneurisms is necessarily very complicated, and as a correct knowledge of that adopted by M. Broca is necessary for the understanding of the precise value he attaches to certain words, his table of the different kinds of aneurism is copied. In order to designate the direct and permanent communication of an artery with a vein, M. Broca makes use of the word *phlebarteria*, a neologism that may well be pardoned, as it shortens much the description of the pathological condition of the parts. The word, however, has been already used by Piorry, in order to designate disease of the pulmonary artery, which M. Broca has omitted to mention.

I. ARTERIAL ANEURISMS.

A. CIRCUMSCRIBED ANEURISMS (aneurisms provided with a sac).

| | | |
|---|---|---|
| 1. The sac is formed by the three tunics uniformly dilated. | TRUE ANEURISM. | |
| 2. The sac is formed only by the external tunic, the two others being torn or destroyed. | EXTERNAL MIXED ANEURISM. | A. *Sacciform.*<br>B. *Fusiform.*<br>C. *Dissecting.* |
| 3. The sac is constituted by a membrane of new formation, the three tunics of the artery being divided at the same time. | FALSE OR ENCYSTED ANEURISM. | A. *Primitive.*<br>B. *Consecutive.* |
| 4. The sac is formed by a cyst developed primitively in the thickness of the arterial wall, and opened consecutively into the cavity of the vessel. | CYSTOGENIC ANEURISM. | |

B. DIFFUSED ANEURISMS (aneurisms deprived of a true sac).

| | |
|---|---|
| 1. Effusion of blood contained in an irregular cavity, and resulting from the tear of an artery. | PRIMITIVE DIFFUSED ANEURISM. |
| 2. Effusion resulting from the rupture of a circumscribed aneurism. | CONSECUTIVE DIFFUSED ANEURISM. |

II. ARTERIO-VENOUS ANEURISM.

A. SIMPLE PHLEBARTERIA, or ANEURISMAL VARIX (arterio-venous communication, with varicose dilatation of the veins, without circumscribed aneurismal tumor).

B. VARICOSE ANEURISMS (phlebarteria, with aneurismal tumor).

| | | |
|---|---|---|
| 1. The sac is formed by the circumscribed dilatation of the vein at the level of the phlebartery. | VARICOSE ANEURISM BY DILATATION. | *Simple*, or communicating with only one vein. *Double*, or communicating with two veins, and forming two sacs (aneurism of Park). |
| 2. The sac is formed by a membrane of new formation. | ENCYSTED VARICOSE OR FALSE CONSECUTIVE VARICOSE ANEURISM. | *Arterial*, the sac situated on the artery (aneurism of Rodrigue). *Venous*, the sac situated on the vein (aneurism of A. Bérard). *Intermediary*, the sac situated between the vein and the artery. |

It will be seen from the table that the tumor due to the dilatation and lengthening of one or more arteries, which become flexuous as veins affected with varix, is not classified as aneurism. The march and structure of the tumor are so different from those of aneurism, that it would be best to keep the name of arterial varix, by which Dupuytren designated it. The erectile tumors, called *anastomotic aneurisms, aneurisms by anastomosis*, and the bloody tumors of bones, are also very properly rejected from the list.

As to the *dissecting* aneurism, Broca limits himself to a simple mention of its existence and of its mode of formation, "as the diagnosis is almost always impossible, and the internal seat excludes the idea of surgical treatment." It has, in fact, only been observed on the large arteries of the trunk of the body, on those, as Erichsen says, "in which the middle coat is highly developed, and the yellow elastic tissues abundant." He says no more, also, of *cystogenic* aneurisms, about which authors disagree, some, as Hodgson, supposing that the arterial cyst, far from being the cause of the aneurism, is, on the contrary, the effect. M. Broca, from some specimens he has seen, is inclined to believe it the cause.

In a therapeutical point of view, the form of the aneurismal sac and the nature of its connections with the artery are much more important than its origin and mode of formation; for it is they that modify the circulation of the blood that passes through them. If pathological anatomy establishes numerous species in the group of arterial aneurisms, pathological physiology, the basis of their rational treatment, is satisfied with but three categories.

1st. Those where the blood has but little or no tendency to coagulate (crateriform aneurisms).

2d. Those where it is disposed to form passive clots (diffused aneurisms).

3d. Those, finally, where all the conditions are favorable to the formation of active clots (sacciform aneurisms and fusiform aneurisms).

Before, however, entering into the consideration of this important portion of the study of aneurism, its pathological physiology, a portion wherein M. Broca, in his fifth chapter, has displayed extraordinary ability, let us glance at some of the preceding chapters of his work, treating, the second of the etiology, the third of the signs, march, and termination, and the fourth of the diagnosis of aneurism. The first chapter, comprising 37 pages, treats of the different kinds of aneurisms, and the classification adopted by M. Broca, having been given, it will not be necessary to enter into any discussion of it; it is the most complete and the most satisfactory that we have met with, after a very extensive examination of works treating of aneurism.

Aneurisms, considered under the head of etiology, are divided into traumatic and spontaneous. It is only under the etiology of the latter class that we find anything to notice. In the table of Crisp (Diseases of Blood-vessels, London, 1847) there are 551 cases of *spontaneous* aneurism, taken *indistinctly* and *exclusively*, internal and external, published in Great Britain from 1785 to 1847. Of 505 of these cases, those in which the age is given, only 5 were persons under twenty years of age. Of these, 3 were in the cranial cavity, 1 in the aorta, and the other, a child of nine years, the aneurism was inguinal. In some remarks made by Mr. Fergusson on a patient, a female, aged 22, brought into his wards at King's College Hospital, that we have met with in the Medical Times and Gazette for December 30, 1854, he states that he had never before met with a popliteal aneurism in so young a subject, and that he imagines hardly a similar instance is to be found in the records of English surgery. There can be no doubt that individuals in the prime of life, and also those who possess the greatest muscular energies, are the most exposed to aneurisms.

Of the cases collected by Crisp, 42 per cent. of the whole number were aortic, 25 per cent. were popliteal, 11 per cent. were femoral, 4½ per cent. were carotid, 4 per cent. were subclavian, 3½ were brachio-cephalic, and 3 per cent. were axillary. It is seen from this collection that popliteal aneurisms form nearly one-half of those that can be treated by surgical means. The cause generally given by authors is the frequency with which the lower extremity undergoes forcible extension. According to Dupuytren or his editor, for (as is often the case in a publication made of the oral lessons of a teacher) it is impossible to decide to whom the remark belongs, one existing cause, not yet mentioned, seems the act of drawing on tight boots. The forced extension of the ham produces pain in the ham, which does not subside for many hours. (Loc. cit. p. 67.) M. Broca attributes this predilection to aneurism for the popliteal artery to an anatomical arrangement of the parts, the importance of which has recently been shown by M. Verneuil, in a memoir "On the True and Primitive Seat of Varices of the Lower Limbs." (See Arch. Générales for 1855.) Contrary to the general opinion, varix always commences in the deep-seated veins of the posterior region of the leg; the venous dilatation stops suddenly at the level of the point where the tibio-peroneal vessels traverse the ring of the soleus, and it is due to the obstacle which this muscular ring opposes to the return of the blood. M. Broca says, "It appears certain that the arterial circulation must also be hindered, and the ring must act on the artery just as upon the veins; and if, on one side it disposes the veins to become varicose, on the other, it disposes the artery to become aneurismatic. It is thus that I explain the frequency of popliteal aneurysms;" p. 51. M. Broca is not the first who has thus explained their frequency, as will be seen by the following passage, which may well be given here, as it also shows the old ideas concerning the etiology of aneurism. It is taken from a translation, published in London, in 1723, of "A Treatise of Chirurgical Operations, according to the Theory and Practice of the most learned and experienced Surgeons in Paris," by Mons. Garengeot. "The true aneurism is commonly occasioned by great agitations, and violent motions in those parts where the muscles exert themselves most. It proceeds also from compressions, occasioned by bony or humoral tumors, as is seen to happen in the intercostal arteries by the *exostoses* of the ribs, in the *clavicula*, the *humerus*, the elbow, and in the artery, which pierces the ligament betwixt the two bones" (of course the popliteal is meant); "'tis plain the blood will rather stop in that place than in the remaining part of the duct; and when this happens, the serosity of that blood stopped must needs separate from it, and pass more easily through the pores of the artery. The membranes of the artery, being imbibed with the serosity, that separates from the blood, grow more pliant, looser, and, consequently, more capable of yielding to the motions of the heart." Garengeot thus explains the mode of action of the compression said to be exerted upon the popliteal artery; and so far he is still in advance of M. Broca, who contents himself with saying that the arterial circulation is interfered with (gênee) by the fibrous ring. The fact is simply this, that the popliteal artery is, of all the branches of the aorta, the one upon which the locomotive apparatus exercises the greatest influence by the almost incessant alternatives of straightening and bending, which it imposes upon it. An alteration of the walls of the artery, which would be without inconvenience in the central portion of the femoral, in the iliac, in the primitive carotid, &c., would expose, to a degree infinitely greater, the popliteal to being torn in one or several places. Pathological changes, which elsewhere would be followed by no serious consequences, would, in the popliteal artery, lead to them.

The etiology of spontaneous aneurism must repose entirely upon the defect of equilibrium between the force of the impulsion of the blood and the resistance of the arterial walls, either that a heart too active is placed at the head of arteries relatively too little resisting, or that a previous alteration has diminished the resistance of the arteries, the heart remaining the same. These alterations in the walls of the arteries have been long noticed, though their effect has often been mistaken. Ambrose Paré, who wrote in 1582, speaking of them says, "And this, by a great foresight of Nature (the handmaiden of the Almighty), constitutes a rampart or stony barrier, lest the hot and boiling blood, full of spirit, should escape and pour out of the coats of the aneurismal or dilated artery." (Observations on Aneurism, selected from the works of the principal writers on that disease, from the earliest periods to the close of the last century. Translated and edited by John E. Erichsen. London. Printed for the Sydenham Society, 1844; p. 97.) At the present day, these alterations are almost universally considered as the cause of aneurism; in fact, we are acquainted with but one author who is not of that opinion. This one is Cruveilhier, who says that he regards the alterations, cretaceous, steatomatous, &c., of the arteries, as a consequence of a disproportion between the force of impulsion of the heart and the arterial resistance; and further still, he maintains that the arterial lesions are very often consecutive to the dilatation. (Loc. cit. vol. ii, p. 800.) With this exception, all other authors agree as far as we have been able to examine them. Miller says, that "for the formation of true aneurism the existence of the steatomatous degeneration of the coats is essential." Erichsen says that aneurism is invariably dependent upon the coats of the artery having been softened, atrophied, and disintegrated by fatty degeneration, and consequently yielding to the concentric pressure of the contained blood. "In the very numerous specimens of dilated arteries that I have examined I have never found one that had not undergone fatty degeneration or atheromatous deposition," is his assertion. (System of Surgery, Brinton's edition, p. 463.) Gross (Elements of Pathological Anatomy, Philadelphia, 1845, p. 237) says that "most of the species of aneurism here enumerated (all the species that are properly classed as aneurisms, for he should have omitted aneurismal varix and nævi materni, which he calls anastomotic aneurisms) are dependent, directly or otherwise, upon an altered or modified state of the arterial tunics." Hasse (An Anatomical Description of the Diseases of the Organs of Circulation and Respiration; printed for the Sydenham Society, 1846, p. 85) says, that in every instance that has come under his notice, the arterial membranes were covered with semi-cartilaginous patches and false membranes, rendered uneven, softened, or partially exulcerated by atheromatous deposits, and extensively ossified. Wedl (edition of the Sydenham Society, p. 229) says, that the elasticity and contractility of the vessels are affected by the fat which accumulates in the walls, as a consequence of the insufficient supply of nutriment—"an expansion takes place at the points more copiously infiltrated with fat, from which, owing to its diminished contractility, the vessel is unable to regain its primitive calibre." Mr. Gulliver (Medico-Chirurgical Transactions, for 1843, p. 90) says that a fatty degeneration of the tunics of the arteries is generally connected with that state of them which is the most frequent cause of aneurism. It being the almost unanimous opinion of medical writers of the present day, that what is called fatty degeneration of their walls is the cause of aneurism, we were not at all prepared to find M. Broca differing from them; and we wonder at it the more, that he gives no reason for so doing. He says (p. 51): "When the repartition of aneurisms in the different arteries of the body is studied, when their great frequency in those near the heart, and when the influence exerted in their production by efforts and fatiguing labor is taken into consideration, no one can refuse to admit that there is something mechanical in the determining cause of aneurism; but this cause would be generally insufficient if it were not favored in its action by a previous alteration of the walls of the arteries, which constitutes the predisposition to aneurisms." This is very well, but when he declares that this previous alteration "is often not appreciable to the sight, and only reveals itself by its effects," that in true aneurisms "generally no deposit is found, no appreciable change of structure in the arterial walls, which are only thinned and weakened," we must feel some astonishment. After some remarks on the usual subjects of climate, alcoholic drinks, syphilis and mercury, M. Broca adds, "Let us say, then, that by the side of the mechanical causes, which determine the formation of spontaneous aneurisms, there are often other predisposing dynamic causes, which act in altering the structure, or in diminishing the resistance of the arterial walls; but let us add, that we are ignorant of the nature of these injurious predispositions."

We are astonished at this chapter on etiology. It being a most important point, we expected at least to find therein what was known, if nothing new. That fatty degeneration, or fatty *substitution*, to use a more correct term, is the great predisposing cause of aneurism, we see no reason to doubt. In every case of fatty substitution the nutrition is modified, its force is diminished, it is rendered more slow. When old age, misery, sickness, or anything comes to interfere with the proper nutrition of the body, fatty substitution is observed; first in all the non-vascular tissues, whose nutrition is manifestly more slow, more easily troubled than that of the vascular. For this reason the elastic tunic of the arteries is one of the first tissues of the body to be affected with fatty granulations. It is thus we should explain the influence of syphilis, of alcoholic drinks, of mercury, of age, and of country. No nation is so much afflicted with aneurism as the Irish, and in none, thanks we believe to the government of England, is there more poverty, more misery, and consequently more drunkenness, nowhere more causes of fatty degeneration of the arterial coats.

In Chapter III, on the signs, march, and termination of aneurisms, we find nothing to remark, at least in that portion concerning arterial aneurisms. Their signs result from their connection with the circulation; they tend always to increase, and abandoned to themselves they terminate fatally, generally by rupture; at the expiration of a time, that varies from a few weeks to many years, some peculiar conditions can bring about their spontaneous cure: these are very rare and will be studied elsewhere.

The portion concerning arterio-venous aneurisms is very clearly written. M. Broca shows how utterly inconsistent with physiology is the hypothesis of Breschet, that the current is alternately from the artery into the vein, and from the vein into the artery. The current is always from the artery into the vein, and hence in this aneurism the bruit-de-souffle, though louder at the arterial diastole, is continuous. This sound is produced at the edges of the orifice in the artery, made to vibrate by the continual, rapid, and abnormal passage of blood. The increase in the length of the limb on the affected side, and the hypertrophy of the pilous system, remarked by M. Broca, are worthy of the most serious attention in a physiological point of view. This aneurism generally remains quiet, above all when in the neck, or in the upper extremities: "Aneurysms increase because the pressure of the blood they receive is superior to the resistance of their walls. Now, in arterio-venous aneurisms, a part of the column of the blood finds an easy issue in the adjacent vein. Consequently, the pressure and the tendency to increase are less than in ordinary aneurisms;" p. 81. But on the other hand they have not so much tendency to get well spontaneously, for fibrous deposits do not take place in them. It results from what precedes, that arterio-venous aneurisms, left to themselves, are much less serious than arterial aneurisms, but it is much more difficult to obtain their obliteration. Experience has shown, in fact, that they are much more rebellious than others, and that they brave the action of the greater part of therapeutical methods.

In Chapter IV, M. Broca considers the diagnosis of aneurism, limiting himself to external aneurisms, or rather to those that form a tumor appreciable to the touch or the sight. This diagnosis is composed of two elements, to distinguish aneurisms from other tumors, and to distinguish different kinds of aneurism from one another.

This diagnosis sometimes offers difficulties, for certain tumors can offer characters similar to those of aneurism, and again, certain aneurisms can be deprived of some of those characters, which serve generally to distinguish them.

When an aneurism is modified by the presence of fibrous clots, so as to resemble very much a solid tumor placed before an arterial trunk, the surgeon, when doubtful, is recommended to exert indirect compression in a permanent manner on the trunk of the artery, above the tumor. "If the tumor be solid, no effect will be produced, but if an aneurysm, on the contrary, at the end of a few days, the sac manifestly becomes smaller, and it is found that in seeking the diagnosis, the treatment has been commenced. In fact, those aneurysms least reducible, whose diagnosis is most difficult, are precisely those that yield most easily and most promptly to indirect compression;" p. 90.

As to abscess, he declares that an abscess need never be taken for an aneurism; that it is always easy to distinguish them. We know of an instance in which a lady in Bordeaux was treated for aneurism, situated in the thorax, and making its way through the anterior wall. Pressure was long applied, in order to support the walls of the sac, and to delay as long as possible, the fatal termination; the lady suffered greatly, when at last the envelopes gave way, and an abscess discharged its contents, to her great relief. In the Medico-Chirurgical Transactions, for 1850, is reported a case "of scrofulous abscess of the anterior mediastinum, communicating with both sides of the chest, the pericardium, and trachea, forming a tumor above the clavicle, and simulating aneurism of the innominate artery, or arch of the aorta." The physician, Dr. Maclachlen, was not deceived, but the case above mentioned shows that the mistake has occurred. The contrary mistake is much more easily committed, and many celebrated surgeons have been so unfortunate as to take an aneurism for an abscess.

A source of error in the diagnosis of aneurisms is pointed out by Verneuil in "a note to aid in the history of aneurisms," printed in the Archives Générales, for May, 1850. It is upon the subject of the momentary suspension of the pulsations in aneurismal tumors. Without being able to account satisfactorily for the phenomenon, he shows that there are certain aneurisms whose pulsations appear and disappear at short intervals. He believes that this is the source of the errors of diagnosis made by celebrated surgeons. He attributes it to "a spasm of the artery, a vital action of the arterial walls in a point more or less distant from the explored part." We may notice, while on this point, that it is to this vital action of the arterial walls that Hodgson attributes the action of indirect compression in the cure of aneurism. At a discussion at the Royal Medical and Chirurgical Society, in London (in 1853), relative to the treatment of aneurism by pressure, the President (Mr. Hodgson) said, "that he believed that pressure, the moderate pressure, now used in the treatment of aneurism, acted by bringing into play the vital contractility residing in the inner portion of the middle or elastic coat of the artery. The vital contractility is brought into action whenever the artery is subject to any irritation." There is, therefore, very high authority in favor of the truth of Verneuil's proposition, that a vital action in the artery can produce the cessation of the pulsation in the aneurism to which it leads.

As a general rule, so far as the treatment is concerned, it is enough, in diagnosticating the seat of the aneurism, to determine what artery holds in dependence the pulsations of the tumor. "When, for instance, pressure on the femoral vessel arrests the pulsation of an aneurism in the popliteal region, there is no necessity for knowing more, in order to give a proper direction to the treatment." "In one case, precision of anatomical diagnosis is of the highest importance, in aneurisms that project at the side of the trachea, immediately above the clavicle. These aneurisms can belong to the aorta, to the brachio-cephalic trunk, to the carotid, or to the subclavian. In the first case, the use of refrigerants and the method of Valsalva can be tried; perhaps even it may be permitted to think of galvano-puncture or of coagulating injections, but no bloody operations can be had recourse to; in the second case, the method of Brasdor, modified by Wardrop, is rigorously indicated; finally, in the last two cases, the carotid could be tied, or the subclavian between the tumor and the capillaries;" (p. 97.) It is seen, how indispensable here is precision of diagnosis, and nevertheless, it is often impossible, because the surgeon is deprived of the principal means of diagnosis,—momentary pressure upon the artery between the tumor and the heart.

The question is still more complex, when the aneurism is arterio-venous; for the tumor is at once in connection with both artery and vein, and we must know not only the artery but also the vein, which is the anatomical seat of the *phlebarteria*. This is doubly important; for the only operation that is admissible in these cases is the ligature of the artery immediately above and immediately below the orifice of the communication. This is, generally, easily found, for there the souffle and thrill are at their maximum, and, moreover, there is often a cicatrix.

Before undertaking the study of the therapeutical methods, before describing how aneurisms can be acted upon, and by what mechanisms their cure can be obtained, M. Broca "believes it to be indispensable to describe, with precision, the phenomena, whose *ensemble* constitutes the pathological physiology of aneurisms, and which sometimes lead to the spontaneous cure of this affection. The indications of treatment, in fact, repose directly upon the knowledge of these phenomena, whose study has been too much neglected until now;" (page 103.)

The two following chapters, the fifth and sixth, the last of the first part of the work, are devoted to these subjects. As the movement of the blood in varicose aneurisms has been described in Chapter III, very little is said of them here; but he promises, "to insist with detail on the physiology of arterial aneurisms,—a study scarcely opened."

On account of the effect of the form of the sac, and the nature of its connections with the artery, upon the circulation of the blood traversing it, M. Broca, as said before, classifies aneurisms as crateriform, sacciform, fusiform, and diffused. Crateriform being but the first degree of the sacciform, the phenomena are like those produced in the latter, only less pronounced; and mentioning them as a distinct species is of no advantage whatever. In the sacciform and fusiform, though the circulation, owing to the position of the sac, is of a different character, yet, in both, it is such as to favor the deposition of what M. Broca calls *active clots.* In the diffused aneurism, the circulation is such as to favor the deposit of what he calls *passive clots.*

In the fifth chapter, sacciform aneurisms, or those communicating with the artery by only one opening, are first considered. Two forces preside over the circulation in the arteries; one, coming from the heart, produces their diastole; the other, due to their elasticity and the *active contraction* of their muscular tunic, produces their systole. The velocity of the circulation is therefore not uniform; at each beat of the heart it is suddenly accelerated. The viscosity of the liquid, and the friction of its molecules against the internal membrane of the vessels, render the movement of the portion nearest the walls slower than the rest, so that the velocity is not only not uniform, but it is not the same for all the molecules. When an opening is made into an artery, the blood flows from it in a continuous and jerking stream; when the opening leads into an aneurismal sac, the blood, unable to escape externally, presents an incessant flux and reflux. The membranous wall of the aneurism possesses a certain elasticity, permitting it to be distended, and to return to its primitive volume when the interior tension is diminished; this retraction is entirely passive; the sac, purely fibrous, or cellulo-fibrous, does not contain any tunic comparable to that which gives to the arteries their power of vital contractility. It results from this, that the renewal of blood, at each pulsation, is very complete at the orifice of the sac, but very feeble in the layers of blood nearest to the internal surface of the aneurismal walls. The blood, in contact with the walls of the sac, is not only more disposed to coagulation from being more stagnant, but also from another cause, namely, the abnormal condition of these walls themselves. The normal contact of the internal membrane of the vessels is necessary for the maintenance of the fluidity of the blood; every other contact exercises an opposite action. In the sacciform aneurism, the lining membrane always constitutes an abnormal contact for the blood; the most immovable layers of this fluid, consequently those that have the greatest tendency to the deposition of clots, are those that must undergo this contact. If the stagnation of the blood were complete, or nearly so, these clots would be *passive;* but as it is not, they are *fibrinous* or *active.* This clot, thus formed, pushed against the wall of the sac by the force of the blood, by degrees being deprived of the serum with which it is imbibed, becomes gradually thinner and condensed. These clots are found formed of layers, because they only form when the sac is of a certain size, and when one layer diminishes it, there is again a cessation, until the sac has enlarged again sufficiently for the circulation to be again sufficiently diminished in velocity for the deposit of clots; (p. 123.) The vascularity of these active clots has been wrongfully denied; and even if not vascular, this is no objection to their vitality; for the hair, the teeth, the cornea, the crystalline lens and the articular cartilages, whose vitality cannot be doubted, are deprived of bloodvessels. "Thus, whatever be the opinion formed of the properties of active clots and of their vitality, it must at least be recognized; and this is an important point in the history of aneurisms, that they do not act as foreign bodies; that they are able to resist chemical and physical actions, which the passive obey; and that, if they did participate in the common life, they would not act differently;" (p. 133.) On account of the resistance, added by the fibrinous layer to that of the sac itself, and of the pressure of the interior liquid having less hold of the internal surface of the sac, from its reduced dimensions, the march of the aneurism is impeded. Thus, these active clots impede the progress of the disease; they always retard it; they can even render it stationary; finally, they can cause it to retrograde, and they can even cure it. As a general rule, however, art must step in; it can do so in many ways; but, as is foreseen, the most advantageous methods are those that favor the formation of these useful fibrinous deposits. It is from misunderstanding the importance of these clots, that most surgeons have a very inexact idea of the indications to be fulfilled in the treatment of aneurism. When a collateral vessel arises from the aneurismal sac, the stagnation of the blood takes place to a less degree than in other aneurisms; but that is its only effect.

Fusiform aneurisms can, to a certain extent, be said to resemble the sacciform, surmounted by a collateral artery. The axis of the aneurism is occupied by the new blood, almost free from mixture; at the periphery, that is, in the neighborhood of the walls, exists the old blood scarcely modified by some of the new. At the periphery, therefore, the blood, finding itself under conditions favorable to coagulation of the fibrine, allows active clots to be deposited on the walls of the sac; (p. 144.)

As to diffused aneurisms, in the *primitive*, as they are entirely deprived of a sac, and as the cellular tissue around the blood does not possess that regular elasticity of the other aneurisms, it is foreseen that this circumstance must greatly modify the circulation; there is every chance of only passive clots being produced. The *consecutive*, having at one part a sac, are between the sacciform and the primitive diffused aneurisms.

In arterio-venous aneurisms, fibrinous clots are very seldom deposited, owing to the small volume of the sac; for the tendency to fibrinous coagulation, other things being equal, is proportional to the capacity of the sac and the slowness of the current. When the tumor is of considerable volume, active clots form perfectly well.

Sometimes local causes are insufficient to explain certain varieties met with in the facility with which these fibrinous layers are produced, and they must be attributed to constitutional causes. Upon these the attention of observers is called, they being supposed to be owing to the proportion of fibrine in the blood. "Some facts that I have had occasion to collect, lead me to believe that the coagulation of the fibrine takes place with the greatest facility in individuals whose constitution presents the characters assigned to the temperament called sanguine;" but he hastens to recognize that these facts are too few to give any great value to the impression he has preserved of them. "The future will perhaps solve this difficult problem; perhaps even the means will be found of modifying the constitution of patients, and of carrying, by an internal treatment, the fibrinous plasticity of their blood to the degree necessary for bringing about the obliteration of the sac, without surgical intervention; this would be the beau ideal of the treatment of aneurisms; but this dream does not seem near being realized;" (p. 151.)

This is the substance of the 48 pages comprised in the chapter on the Pathological Physiology of Aneurisms, the most important chapter in the work, insomuch as it is at the foundation of the judgment he passes upon all the methods of treatment discussed in the second part of the book, and also of the remaining chapter of the first, on the spontaneous cure of aneurism. There is nothing so important in the history of aneurism, as the study of the nature and source of the fibrinous layers found in aneurismal sacs, and also of the constitutional condition that favors their formation. The hypothesis that the sanguine temperament is favorable to fibrinous deposits, is the only new thing in the whole chapter, if it be new; in every medical work we find the nature and source of the fibrinous layers indicated. Gross says (loc. cit. p. 237): "In the sacculated variety of the disease, as well as in some of the rest, one of the earliest effects consequent on the development of the tumor, is the deposition of the fibrine of the blood, as this fluid sweeps over its inner surface." Wedl (Pathological Anat., published by the Sydenham Society, London, 1855, p. 229) says: "Now the stream of blood must be delayed in the dilated portions of the vessel, or may be even partially arrested in some of the hollows, or perhaps entirely stopped, when a coagulum is formed, by which the canal of the vessel is obliterated, and which undergoes several morphological changes." The same observer examined an old coagulum contained in a very large aneurismal sac of the aorta, and found it to consist "of several fibrinous layers." Hasse (loc. cit. p. 90) says: "These fibrinous formations, so highly important as regards the pathology and treatment of aneurism, are, in all probability, deposited directly from the more or less stagnant blood within the sac; similar fibrinous layers surround the extravasation in diffuse spurious aneurism. Kreysig may possibly be correct in supposing a concurrent inflammatory condition of the membranes to co-operate in their development; but Schœnlein's idea of their being the essential constituent of aneurism, and of a fungoid carcinomatous nature, is wholly untenable." Hughes, in his observations on the fibrinous formations in the heart (Medical and Surgical Monographs, Philadelphia, 1840) says: "That coagulation of the blood may occur in the vessels in the living body, is a fact clearly proved by the results of disease, and by experiments in the lower animals. That this coagulation generally depends upon one of two causes, viz., inflammation or other disease of the vessels themselves, or considerable retardation of the motion of the blood, will, I presume, be generally admitted; the coagula found in aneurismal sacs may perhaps be dependent upon both causes combined. But perfect rest is not necessary to the coagulation of the blood, either in the vessels or when removed from the body, for it is, I conceive, impossible to suppose that perfect rest can exist in the fluid contents of an aneurismal sac, when coagulation first occurs, or when each successive fibrinous layer is deposited. Comparative rest, or a considerable retardation of the onward current of the blood, is sufficient to induce coagulation, and upon this circumstance the operation for aneurism, particularly when performed on the distal side, is dependent for success." We will add also, the following extracts from Cruveilhier, the work before cited: "The clots appear destined to protect the walls against the direct shock of the blood, and consequently to prevent their rupture. They have been erroneously considered as aiding by their pressure to accelerate the rupture of aneurismal sacs, for the blood-clots are not susceptible of spontaneous alteration;" (p. 775.) "If blood-clots could organize themselves anywhere, it would be in aneurismal sacs, for nowhere can the coagulated blood find itself in conditions more favorable for organization. Now blood-clots are never organized. These clots are not organized; it is crystallized fibrine. They increase by juxtaposition, and even by intussusception; they are in themselves unalterable." "This adherence of the clots to the sac, and of the layers of the clots to each other, is not vital; it is the consequence of the physical properties of the coagulated blood;" (p. 773.) "It has been supposed that the formation of blood-clots was due to the stasis of the blood in the aneurismal sacs, and it was said, the blood-clots do not form in vessels simply dilated, because the circulation is still free in the parts which are the seat of the dilatation, but they are formed in aneurisms where there is rupture of the coats, on account of the stasis. But the cause presiding over the formation of blood-clots in aneurisms is the same as that presiding over the formation of blood-clots in all the vascular canals, to wit, adhesive inflammation;" (p. 774.) "What prevents this mode of cure (by blood-clots) from being definite, is the ulterior changes that take place in the sac filled with clots, these changes being owing to the impulse of the blood; hence, diminish this, and the cure becomes definite. Art can step in, by diminishing as much as possible the activity of the cardiac circulation by the alimentary regimen. Refrigerants, ice, digitalis, &c., these are the means proper to consolidate the cure;" (p. 802.) These citations are quite sufficiently numerous; all authors of the present day, except one German, Schœnlein, and with his much learning, it is not wonderful that the German should sometimes be made mad, agree as to the effect of these clots, as to their utility as protective against the force of the blood. With regard to the causes favorable to their formation, and also with regard to their nature, it is also seen that there are differences of opinion. With some the retardation of the current in the sac is sufficient to cause the formation of the clots, with others adhesive inflammation of the vessel conduces to this, with others this inflammation is indispensable. With some the clots are merely condensed coagula, with others they are deposited fibrine; with some it is crystallized fibrine, and never can be organized; with M. Broca it is vascularized, in other words, organized. With the exception of the point regarding the vitality of the deposits, about which the Irish author says nothing, M. Broca agrees in every point with Bellingham. (Observations on Aneurism, and its treatment by compression. London, 1847.) Bellingham says (loc. cit. p. 136), "These familiar facts all tend to prove—

"1st. That Nature herself sets up a process by which, under favorable circumstances, the cure of aneurism is effected.

"2d. That the mode in which she effects this, always in internal aneurism, and frequently in external aneurism, is by the deposition of the fibrine from the blood in the sac of the aneurism, until it becomes filled.

"3d. That the fibrine in such cases is deposited in regular concentric laminæ, the oldest or first formed next the sac, those most recently formed nearest the centre.

"4th. That a current of blood through the aneurismal sac is a necessary agent in bringing about this result.

"5th. That every obstruction to the current by which its velocity and amount are diminished, will accelerate the deposition of fibrine in the aneurismal sac."

On this subject, M. Broca says, "M. Bellingham has indicated the true origin of fibrinous clots, above all, he has unveiled the conditions in favor of which they are deposited in aneurisms. He is far from having exhausted all the questions that attach themselves to this interesting study; but he has at least furnished precious *données*, which permit us now to establish on solid foundations the pathological anatomy of aneurismal tumors. I have much profited by the reading of this work, which I cannot too much recommend to the attention of surgeons;" (p. 127.) There is no more incontestable statement in the whole of this large book, than the last, that he has much profited by Bellingham's work. Not only on points connected with the pathology of aneurisms, is he much indebted to it, but also, where it is at first less evident, on the historical points connected with the subject. It is, of course, impossible to prove our assertion in the limits allotted to a review, but we are sure that any one acquainted with the two works, will at once acquiesce in its truth, that there is nothing of any value in the work of M. Broca, which is not taken from that of Bellingham.

Bellingham distinguishes between a coagulum of the blood and a fibrinous deposition, but does not speak of the latter as *organized*. M. Broca, giving exactly the same mode of formation, maintains that they are. No one, at the present time, has any right to maintain such a proposition; when he says that they are organized, and, at the same time, that they are formed by successive deposits from the circulating fluid, he maintains what, in his language, is called a *bêtise*.

The subject of the origin of these layers, formed in aneurismal sacs, is a very unsettled one. Only one thing about them is positively determined, namely, that for furthering their formation, the current of blood traversing the sac should be diminished. That they adhere closely to the sac, and to each other, and that they do not act as foreign bodies, shows, to our mind, that they are organized; and that they are organized shows that they are not formed as Bellingham, and M. Broca after him, supposes. They make a distinction between a coagulum and the layers of fibrine, but their explanation that the blood first coagulates and then forms a deposit of fibrine, which adheres to the walls of the sac, is not only inconsistent with our ideas of pathology, but we believe that it fails to account for many of the facts observed in the progress and cure of aneurisms.

As must be noticed in the citations given from authors, speaking of these fibrinous layers, or active blood-clots, or deposits of fibrine, whatever they may chance to be named, an inflammation is supposed by some to coincide, some say most probably, others say necessarily, with their formation. This M. Broca, fortunately consistent with himself, is unable to believe. His anatomy shows him no vascularity in the middle and internal tunics of the artery, and, therefore, they cannot inflame. He sees examples of their inflammation everywhere, he sees organized deposits in aneurismal sacs, whose walls are formed of the arterial tunics yet unbroken, but his anatomy declaring it to be impossible, it does not exist. Just as the Italian philosophers, who saw what Galileo showed to them, but declared it was not so, because Aristotle had said that it was impossible.

We believe ourselves that this striated substance contained in the sac is an effusion of lymph, poured out by the sac itself. We believe this because such a doctrine is in accordance with the nature and properties of the substance itself; it is in accordance with the process adopted by nature in other parts, or in similar parts under other circumstances. It offers, moreover, a ready solution of the changes observed during the course of the disease, and leads to a due appreciation of the various remedies and means proposed for cure.

This was the doctrine professed by Colles, and it may be found in an article on aneurismal sacs by his son, published in the Dublin Quarterly for 1856. He says, on the subject of these fibrinous layers: "This theory that the circulating fluid is the cause of the increase of the disease, by the impulse, and at the same time of its cure by a deposit of fibrine as it passes, leads to uncertainty and vacillation in treatment; at one time the object being, by bleeding and starvation, to diminish the quantity of blood, and at another, by nutritious diet, to increase the fibrinous element." "On the contrary, if we consider that these layers of lymph are formed by the sac, it would smooth away most difficulties; it would be analogous to what we observe to occur in other parts, whether in solutions of continuity, in extravasations of blood, or in the closing of divided arteries. We can now understand the origin and progress of the disease; we have the blood of the circulation driven at each pulsation by the force of the heart and the action of the arteries, and tending to cause further dilatation of the sac; and to oppose this, we have the sac thickening and strengthening itself by the effusion of lymph, layer after layer; but still we find the sac compelled gradually to give way, and increase in size, because, we know, it requires all the healthy powers of the inner dense, firm coats of the vessel to resist this force, which we can hardly expect to meet in newly-formed layers of lymph, no matter how thick." "In treatment we should have two objects in view, to oppose, to lessen, or entirely stop the current of blood which distends the sac at each pulsation; and at the same time to support the sac in its efforts to oppose this. The pressure should be moderate, for the object is only to give support."

We believe, therefore, the opinions of M. Broca on the origin of these deposits to be erroneous; and when he goes any further than Bellingham, and pronounces upon their nature, though he is right in believing them organized, yet that in so doing he is inconsistent with himself. In respect to the constitutional predisposition favoring their production, although he may have more pleasing dreams, he is not a whit in advance of Hodgson, who, in his celebrated Treatise on the Diseases of Arteries and Veins, says: "It is probable that some particular articles of diet may contribute more than others to promote the formation of coagulum; but on this subject experience has not hitherto furnished any decisive indications." (Edition printed in London, 1815, p. 162.) The opinions concerning the tendency to the formation of fibrine and the causes influencing its proportionate quantity in the blood are so varying and conflicting, that it is best to work seriously to throwing light upon the subject, and dreaming is scarcely to be permitted. M. Broca scarcely notices the question, but it is one to which, on account of its predominant importance, too much attention cannot be turned. The following opinion is the one upon which we would base our treatment of aneurism, were we convinced that fibrinous layers were deposited from the circulating fluid in aneurismal sacs: "An augmented proportion of fibrine in the blood can be taken as an indication only of increased labor and waste in certain elements of the body, not of an increased development in the resources and nutrition of the blood." (General Pathology, by John Simon; American edition; Philadelphia, 1852; p. 43.)

The sixth chapter, comprising 54 pages, is devoted to the spontaneous cure of aneurism.

It was long known that aneurisms may get well spontaneously; but Hodgson, as M. Broca says, was the first to assemble the scattered documents, and to make them the object of a dogmatic study. Aneurism is an incurable disease at any period, in this sense, that the arteries have undergone a morbid dilatation with or without destruction of the arterial tunics, and can never retake their primitive condition. But the walls of the arteries may be so strengthened by the deposit of the fibrinous layers above spoken of, that they are able to resist the distending impulse of the blood, or again, in consequence of a violent inflammation of the sac and the neighboring parts, terminating in suppuration and gangrene, the obliteration of the sac may be effected. (See Cruveilhier, loc. cit. p. 796.) Hodgson records three modes of spontaneous cure. 1st. By the gangrene of the tumor. 2d. By the pressure the sac exercises on the artery, causing the walls to adhere. 3d. By the gradual deposit of fibrine until the sac is obliterated, with or without obliteration of the adjacent artery. The second mode is, according to Cruveilhier, rather rational than demonstrated by experience. If it does occur, it is, as the authors of the Compendium say (Vol. II, p. 98), rather by diminishing the force of the circulation, and thus favoring the formation of fibrinous deposits, either from the circulating blood, as most authors believe, or from the sac, as we believe ourselves. There is an observation on this pressure exercised by the sac, made by M. Broca (p. 159), which is very just, namely, "that if the sac does compress the artery, it must be excited to do so by a force coming from the artery, which force is diminished at once by this compression." In other words, a cause produces an effect, which effect diminishes the cause producing it, as it is produced. Such causes are never very efficacious. Crisp (loc. cit. p. 178) says: The spontaneous cure may be effected: first, by the pressure of the sac upon the artery; secondly, by inflammation and deposits of lymph on the cardiac side of the vessel; thirdly, by the distension of the sac with coagulated blood; and fourthly, by sloughing of the sac and profuse hemorrhage, so as to produce syncope and coagulation of blood in the artery. Gross (loc. cit. p. 238) gives five modes: 1st. The most common process is the formation of fibrinous concretions, by which the whole interior of the sac, with the exception of a small narrow channel, is gradually filled up; the affected artery itself remaining pervious, and carrying on the circulation. 2d. By inflammation, accompanied by the coagulation of the blood in the sac and in the artery, and followed by profuse suppuration. 3d. By the development of tumors in the course of the artery, their pressure and their leading to the coagulation of the contents; the tumor itself may overlap and block up the artery. 4th. By gangrene and sloughing. 5th. By a clot of blood becoming detached and blocking up the opening of communication between the artery and the sac. Nélaton (loc. cit. p. 451) gives six modes of cure, but they are effectively the same as the five of Gross; the second and third being the same as the first of the latter author, modified by the form of the sac, and its situation with regard to the artery. The mode of cure by obliteration of the vessel on the cardiac side by inflammation, M. Broca insists to be erroneous, at very great length. He has some sturdy adversaries: Hodgson, Astley Cooper, and Crisp, being among the number. He insists at such length for two reasons: first, that it is false; second, that it has exerted a deplorable influence upon the treatment of aneurisms. The reason, the sole one, he can give for its falsity is, that it is in opposition with what we know nowadays of the pathology of arteries. Here M. Broca is wrong. It may be in opposition to a peculiar pathology founded upon peculiar anatomical views, but it is not in opposition with the true pathology of arteries. The two inner coats of the arteries not having vessels, they cannot inflame, he would say; but we have referred to this error before. "In acute idiopathic arteritis, the plastic matter that is forced out by the inflamed patch in the artery appears to be deposited in four different ways. 1st. It may be deposited upon, and become adherent to the lining membrane of that portion of the vessel which is inflamed. In such cases as these the artery will be found to be plugged up, more or less completely, by firm, tough, leathery, buff-colored fibrine, which is so closely adherent to its lining membrane, as to be stripped off with difficulty from it." (Transactions of the Pathological Society, London, 1856, p. 173.) This is taken from a report made by Erichsen, and to doubt the evidence of the senses from preconceived ideas on a point of this kind is not at all permissible. The second reason is a true one: it has exercised a deplorable influence on the treatment of aneurisms, to suppose that inflammation of the artery produced its obliteration, but it was only because it was supposed that the obliteration of the artery on the cardiac side was necessary for the cure of the sac. Here is where the error lay, and not in the belief that an arterial vessel could be made to inflame, and that its inflammation could produce its obliteration. "Because it was believed that the method of indirect compression acted by obliterating the artery in the point where the pad was applied, it was concluded that it must act with great force in order to rub the arterial walls, so as to put them in intimate contact, in order to make them inflame and adhere together. Therefore, violent machines were employed, serious accidents were excited, and this precious method was so compromised that it disappeared from practice for nearly twenty years, and it still struggles against the recollection of its former failures;" (p. 158.) This is true, but for that it is not erroneous to believe that an artery may inflame; a truth may be very falsely applied, but for that it is not false itself.

Of all the different modes of cure, given by authors, some are then rejected by M. Broca, and the rest are reduced to two, which he studies in succession. They are: 1st. Inflammation; 2d. Fibrinous coagulation.

"Inflammation is more often injurious than beneficial; it gives rise, however, to some *accidental spontaneous cures.* Fibrinous coagulation is the result of a natural process, always salutary, and always durable. It is to it that are due *natural spontaneous cures;*" (p. 161.)

The spontaneous obliteration of the aneurismal arteries of the limbs has been several times observed, in consequence of violent inflammation that had attacked the aneurismal sac, and the surrounding parts, and that had terminated by suppuration or by gangrene. Most generally the patient dies, worn out, before the completion of this process; sometimes even he dies of hemorrhage, when the superior and inferior ends of the artery have not been obliterated, when the inflammation caused gangrene and suppuration of the sac. Erichsen says: "In some extremely rare cases it has happened that the inflammation thus set up occludes that portion of the artery that communicates with the aneurismal sac, and that thus a spontaneous cure results by its obstruction, and by the adhesive inflammation of the vessel;" (p. 471.) Bellingham speaks of the inflammation caused by the pressure of more or less coagulated blood in the sac, and of the rarity with which this inflammation terminates favorably as the great danger of the presence of a coagulum in the sac. (Loc. cit. p. 144, &c.)

This is the substance of what M. Broca has written on the subject: The spontaneous cure by inflammation can arise in two very different conditions; as the sac is still permeable to the blood, or is already entirely obliterated by passive clots. In the latter case, inflammation is the consequence and not the cause of the obliteration; it only impedes a cure, which without it would be almost certain. The conditions under which it is manifested, and the influence it exercises on the tumor, are indicated further on; here, M. Broca does not occupy himself with them, for he is only to speak of inflammation arising in a sac, when the blood still traverses it, when pulsations are still felt therein, and when reinforced or not by some fibrinous clots, it communicates freely with the artery. The causes of this inflammation may be a contusion, a violent effort, that too rapidly distended the sac, &c. It is not impossible for the inflammation to commence directly on the membrane of the sac; but, most generally, it is developed in the neighboring tissues, and then propagates itself to the aneurism. In the examination of the effects of this pathological process, the symptoms common to most inflammations, such as pain, heat, redness, swelling, &c., are very properly omitted; the special phenomena, which result from the presence of the aneurism, are alone considered. The tumor becomes hardened, and the pulsations diminished, owing, as examination shows, to the formation of a mass of clots, which fills more or less completely the cavity of the sac. The mechanism of the formation of these clots it is not easy to discover; it is perfectly true, that the contact of the inflamed membrane favors the coagulation of the blood; but the determination of the fact does not give its explanation. The nature of these inflammatory clots is of much more importance; and when they are examined after the incision of the sac, or after it has spontaneously opened, they are found to be soft and friable, that is, *passive.* This being determined, three cases can present themselves: the consequences of the inflammation vary according as it terminates by resolution, as it ends in suppuration, or as it is complicated with gangrene. In these three cases also, the termination can be less favorable, either that the aneurism persists, or that the patient succumbs.

As suppuration and gangrene are more common than resolution, they are first studied. The suppuration may be in the sac, or in the cellular tissue around it; generally it is in both points at once. The sac at last opens, and the patient dies more or less promptly from the hemorrhage. M. Broca says: "It is seen how much this termination by suppuration is to be dreaded, which scarcely compensates by a few chances of definite cure, the chances far more numerous of death more or less prompt." The few chances of definite cure are not given; they can only be those of the adhesive inflammation of the walls of the artery, and that for him is impossible. The inflammation can terminate in, or rather be *complicated* with gangrene; for mortification is rarely due simply to excess of inflammation. This is the way it generally occurs: the tumor, in growing larger, presses the inflamed skin; and parts inflamed, when pressed upon, readily mortify; the gangrene comprehends the skin and the walls of the sac, which have become adherent; when the sac is detached, the contents of the aneurism freely escape. Gangrene is not then a mode of cure, it is an accident that depends upon the inflammation. Gangrene prevents the cure of many aneurisms, that inflammation, without its occurrence, would cure, and never adds the least favorable chance to those already existing. The idea of curing aneurism by gangrene, is due to an erroneous interpretation of the fact, that in gangrened limbs, the arteries are found obliterated; it is the cause of the gangrene, not the effect; (p. 169.) The hypothesis of Crisp, that the sac, in detaching itself, gives rise to a loss of blood followed by syncope, which lasts so long that the blood in the artery is coagulated, and the patient cured, is answered by M. Broca, by the question if a simple incision into the sac would cure the patient? Whenever the surgeon has involuntarily made the experiment, the patient has died, with or without syncope; (p. 170.) Independently of the local gangrene taking place about the aneurism itself, in consequence of the inflammation, there is another kind, the sphacelation of the limb below it, from the passive coagulation provoked by the inflammation, and not limited to the sac. All cures of aneurism may be followed by this, but the cure by passive clots is most liable to it, because those clots form so rapidly, that the collaterals have not time to dilate, to gradually become developed. And, finally, the inflammation can terminate by *resolution.* The symptoms of aneurism disappear—in the most fortunate cases, the tumor has completely ceased to pulsate—but the patient is not safe, on account of the nature of the coagulation, which is constituted by passive clots. The different consequences of the obliteration by passive clots must be studied; (p. 172.)

Whenever the obliteration of an aneurism is due to passive clots, a relapse must be feared; and also the consecutive inflammation and rupture of the sac. As they are not organizable, the current of the blood can break them up and wash them away. Generally, however, aneurisms obliterated by inflammation remain permanently impermeable, because the passive clot is soon sustained by fibrinous deposits. This deposit, the fibrinous layer, which is found in these cases when an opportunity is given to examine them, did not exist before the coagulation of the contents of the aneurism, for it was perfectly reducible; it could not have separated from the coagulum, for the fibrine in the blood is but the three-thousandth of the mass; it must, therefore, have come from the sac. Unable to account for it in any other way, M. Broca says, "I think then, that the aneurismal sac, in contact with the decomposed blood it incloses, secreted slowly a mass of plastic lymph;" (p. 175.) He is here forced to conclude, it is indeed impossible to deny such a conclusion under the circumstances, that the sac can, and does sometimes secrete plastic lymph.

After obliteration by passive clots, consecutive inflammation is always to be feared. Its march and rapidity are variable; sometimes it is limited to the walls of the sac, and sometimes it is propagated to the peri-aneurismal tissues. The epoch until which this obliteration exposes to consecutive inflammation, it is impossible to decide; one case is given in which the patient died in consequence twenty-two years afterwards; (p. 178.) It is a mode of cure full of dangers, and chance alone decides the fate of the patient; therefore, M. Broca has given to it the name of *accidental.*

The second method of spontaneous cure in aneurism, that by fibrinous coagulation, is due to a natural process, already studied in the preceding chapter. The fibrinous layers gradually increase in thickness, and the cavity of the sac gradually diminishes until cure results. This sometimes takes place without the patient being aware of it. Sometimes, at the moment of obliteration, the patient experiences a sharp burning pain, lasting for some hours. This pain, which it is difficult to account for, might be explained, according to M. Broca, by the pressure exerted by the tumor recently hardened, upon the nervous threads surrounding it. This pain is attributed by Tuffnell, with more probability, to the dilatation of the artery accompanying the communicans peronei nerve; this being one of the three collaterals which he has noticed in popliteal aneurisms as undergoing most evident enlargement. We have never read of this pain as occurring anywhere but in the lower extremity. "The natural deposit of fibrinous clots is effected by favor of the *incomplete* stagnation of the blood in the peripheric layers; more complete, passive clots are precipitated; less pronounced, there is no coagulation;" (p. 181.) One condition favorable to their formation is then known, and thus the situation of the orifice, the peculiar disposition of the sac, &c., can sometimes explain their presence. "But in the majority of cases, this cure is dependent upon a more general cause, a peculiar plasticity of the blood, the nature of which is entirely unknown;" (p. 182.) The origin, the structure, and physical properties of these clots having already been considered, M. Broca has now only to study the phenomena that follow the fibrinous obliteration of aneurisms. As soon as the tumor has ceased to pulsate, it is found to be very hard; it then becomes rapidly smaller, a few days being sufficient to reduce it to one-half or one-fourth of its former volume. After some time, this diminution in size is effected more slowly; with some patients, a small, hard tumor remains definitely; with others, it is entirely absorbed, only a small flat disc being left; (p. 184.)

This striking phenomenon, the rapid decrease of the sac, M. Broca supposes to be owing to a property inherent in the substance itself of the active clots, and that this property plays an important part in the natural cure of aneurisms. "They have a natural tendency to contract together upon themselves; when thin, this tendency is counterbalanced by the impulse of the blood, but when their thickness has increased at the expense of the cavity which they circumscribe, their retractility, which increases in proportion, ends by becoming superior to the force that is tending to push them back." "When the tumor is entirely obliterated, it is again the retraction of the fibrinous clots that causes it to decrease in size. These clots, in fact, are not composed exclusively of pure fibrine; they inclose a notable quantity of water. In retracting, they dry themselves, so to speak, they expel the water with which they are impregnated; this transudes through the membrane of the sac, and is soon absorbed. The contents of the aneurism thus become more and more condensed;" (p. 185.) When the clots are reduced to their fibrine alone, this retraction and this condensation are sometimes arrested, and the parts remain definitely in this condition; at others, this fibrine itself is gradually absorbed. The whole of M. Broca's views on this subject, as may be judged from what we have said before on the formation of these fibrinous deposits, are in our opinion erroneous; we even feel tempted to use another word, when we read of these clots squeezing the water out of themselves through the walls of an aneurismal sac, when it is to be absorbed. How in the name of common sense could such a phenomenon occur! Admitting that the clots, composed, according to himself, of coagulated fibrine, were saturated with water, if they underwent pressure between the blood and the walls of the sac, is it reasonable to suppose that the water, thus expressed, would pass through the solid walls of the sac, and not into the circulating fluid? M. Broca believes these clots to be organized (p. 131), and yet they are not, according to himself, even formed of pure fibrine deposited from the blood. He does not believe them to be pure fibrine, and yet they have such inherent contractile force as to squeeze out of themselves, through the firm membranes composing an aneurismal sac, such a quantity of water as to diminish the whole mass of the tumor one-half or three-fourths. This is, at least, *bizarre.*

We believe, ourselves, not only that the fibrinous layers by which aneurisms are cured are secreted by the walls of the sac, but we also believe, that the rapid diminution in the size of the tumor is due to the walls of the sac, owing to their vital contractility, which is allowed to act when the force of the circulation is diminished. For this opinion, at least, we have most excellent authority; Erichsen says: "Though I have stated generally that aneurisms are cured by the deposit of laminated fibrine, I think this remark ought to be confined to the common sacculated form of the disease. In the tubular variety (or the fusiform), which is certainly of far less frequent occurrence in the extremities, the cure of the aneurism takes place by contraction of the sac, and by its becoming filled with fibrine in a somewhat irregular manner." (System of Surgery, p. 419.) We notice also that Paget, who, it is to be remarked, does not speak of aneurisms in his Surgical Pathology, is of the opinion, that the effects of continued obstruction of the trunk would be the gradual contraction of the dilated portion, rather than its filling up with clots. (Amer. Jour. of Med. Sci. for Oct. 1851, p. 502.) This contraction is thus explained by Hodgson: "The contraction of the sac and the artery seems dependent upon a law in the vascular system, whereby it appears that when the current of blood is obstructed in its passage, or is conveyed in a diminished stream, the vessel gradually contracts in diameter until it corresponds with the current which passes through it, or its cavity is finally obstructed. Thus the same process of obliteration takes place in the umbilical vessels and the ductus arteriosus soon after birth, as in an artery through which the stream of blood is obstructed by the application of a ligature." (Loc. cit. p. 145.)

The influence exerted on the general circulation of the limb by the fibrinous obliteration of an aneurism, is next spoken of. By this obliteration the artery is sometimes obliterated at the same time as the sac, and sometimes the sac alone is filled. The two dispositions must be studied separately, for one endangers the circulation, while the other restores, as much as is possible, the circulation to the physiological condition. Singular as it seems, the obliteration of the artery is so much the more common, that Scarpa thought it a *sine quâ non.* He says: "It is a certain and incontrovertible fact in practical surgery, that a complete and truly *radical* cure of aneurism cannot be obtained in whatever part of the body this tumor is situated, unless the ulcerated, lacerated, or wounded artery, from which the aneurism is derived, is, by the assistance of nature, or nature combined with art, obliterated and converted into a perfectly solid ligamentous substance for a certain space above and below the place of the ulceration, laceration, or wound." (Treatise on Aneurism, Edinburgh, 1808, p. 188.) This obliteration of the artery, it may also be stated here, is believed by him to be caused by the sides of the vessel adhering by adhesive inflammation. (Loc. cit. p. 196.)

This continuation of the process of fibrinous deposit, is a very difficult point for M. Broca: "For, it seems, as if the coagulation of the fibrine must stop, when the diminution in the velocity of the current, which plays so great a part in the formation of fibrinous deposits, no longer exists;" (page 187.) He overcomes this difficulty, by saying, that "the diminution of the velocity is not the only cause of fibrinous coagulation: the contact of a foreign body is sufficient; the blood, constantly coming in contact with the fibrinous clots, is deposited." If this be true, we see no reason, why the deposition does not go on until all the arterial tubes are obliterated, or until the patient dies. M. Broca said (page 133), that these fibrinous clots do not act as foreign bodies; and here, to support his hypothesis, or rather the hypothesis he has adopted as his own, he bases his reasoning upon such action. It is impossible to discuss all the points in which we differ from M. Broca; but here it seems to us, that the theory of Scarpa is correct, and that the vessel is obliterated by adhesive inflammation.

At the termination of this study of the spontaneous cure of aneurism, M. Broca says: "These notions, which the study of aneurisms furnish us, when abandoned to the resources alone of the organism, are of the greatest importance; they show us, so to speak, with the finger, the object the surgeon should propose to himself in the treatment of aneurisms. He ought to beware of imitating accidental cures; he ought, on the contrary, to have always in view to imitate, or rather to facilitate, the natural cure. All the methods which have for results to provoke the inflammation of the aneurism, or to cause passive clots to be precipitated within it, are by this very thing inferior, in the point and view of pathological anatomy, to those that act simply by favoring the deposit of active clots.

"To obtain active clots! Such is the great idea, that should rule the thoughts of the surgeon in the treatment of aneurism. But, what are the ways that can lead to this end? The mind discovers two.

"Fibrinous coagulation, in fine, depends upon two principal causes: a general cause, the plasticity of the blood; and a local cause, the diminution of the velocity of the circulation. Two indications result from this.

"Of these two indications, the first is, at the present time, and will be for a long time, perhaps, beyond the reach of our means. We do not know how to exercise any influence on these conditions of plasticity whose nature is, moreover, unknown to us.

"But, it depends upon us to fulfil the second indication. We know the mechanical conditions of the circulation, and we have a hold upon them. In the present condition of the science, our efforts ought then to be grouped around this idea: *To diminish the flow of the blood in the diseased artery.* The best therapeutical method will be that which will attain this end with most certainty and least harm.

"Now that we have a compass to direct us in the choice of the methods, we shall be able to study understandingly the numerous means that have been imagined for the cure of aneurisms."

Though we do not agree with M. Broca, as to the origin of the fibrinous clots by which the aneurismal sac is obliterated, yet we agree with him entirely, as to its being the object of the surgeon to cause, or aid, in their production; and, moreover, that, in order to do so, the surgeon must *diminish the flow of the blood in the diseased artery.* As this is, generally, the basis upon which he judges the methods of treatment discussed in the second part of his work, the one whose examination it is now our duty to undertake, it is an easier and more agreeable task for us to enter upon it. W. F. A.

(To be continued.)

